# Physical Mechanism of One-Photon Absorption, Two-Photon Absorption, and Electron Circular Dichroism of 1,3,5 Triazine Derivatives Based on Molecular Planarity

**DOI:** 10.3390/molecules28124700

**Published:** 2023-06-11

**Authors:** Xiangtao Chen, Xiaoyan Shi, Fuming Yang, Xiqing Zhang, Rui Dai, Yan Jia, Ningte Yan, Sixuan Li, Zihan Wang, Zhongzhu Liang

**Affiliations:** Center for Advanced Optoelectronic Functional Materials Research and Key Laboratory of UV Light-Emitting Materials and Technology of Ministry of Education, College of Physics, Northeast Normal University, Changchun 130024, China; chenxt@nenu.edu.cn (X.C.);

**Keywords:** molecular planarity, 1,3,5 triazine derivatives, OPA, TPA, ICT, dipole moment of molecular fragment, electrostatic potential

## Abstract

We provide a method to regulate intramolecular charge transfer (ICT) through distorting fragment dipole moments based on molecular planarity and intuitively investigate the physical mechanisms of one-photon absorption (OPA), two-photon absorption (TPA), and electron circular dichroism (ECD) properties of the multichain 1,3,5 triazine derivatives o-Br-TRZ, m-Br-TRZ, and p-Br-TRZ containing three bromobiphenyl units. As the position of the C–Br bond on the branch chain becomes farther away, the molecular planarity is weakened, with the position of charge transfer (CT) on the branch chain of bromobiphenyl changing. The excitation energy of the excited states decreases, which leads to the redshift of the OPA spectrum of 1,3,5-triazine derivatives. The decrease in molecular plane results in a change in the magnitude and direction of the molecular dipole moment on the bromobiphenyl branch chain, which weakens the intramolecular electrostatic interaction of bromobiphenyl branch chain 1,3,5-triazine derivatives and weakens the charge transfer excitation of the second step transition in TPA, leading to an increase in the enhanced absorption cross-section. Furthermore, molecular planarity can also induce and regulate chiral optical activity through changing the direction of the transition magnetic dipole moment. Our visualization method helps to reveal the physical mechanism of TPA cross-sections generated via third-order nonlinear optical materials in photoinduced CT, which is of great significance for the design of large TPA molecules.

## 1. Introduction

The world’s first ruby laser marked the beginning of the study of nonlinear optical properties [1], and the upconversion fluorescence experiment confirms and promotes the development of third-order nonlinear materials [2,3]. In recent years, third-order nonlinear optical polymer materials have become a research hotspot due to their great application potential in the fields of photoelectric devices, image processing, all-optical switches, and optical storage and memory systems [3,4,5,6].

Traditional one-photon absorption (OPA) uses ultraviolet or visible light to excite fluorescent molecules, which can only happen if the photon energy matches the energy difference between the ground state and the excited state [7]. Two-photon absorption (TPA) uses a light source with nearly twice the wavelength of linear absorption of the sample to excite the sample, so that it directly absorbs two photons through an intermediate state to transition from the ground state to the excited state [8]. The difference is that the wavelength of excited light used in TPA is twice that of OPA. Thus, because the photon energy is low, it causes less damage to the material under high penetration [9]. TPA has great application potential in biological imaging, photodynamic therapy, optical limiting, photochemical reactions, microelectronic technology, and many other fields due to its unique photoresponse characteristics, nonlinear properties, and extremely high spatial resolution [10,11,12,13,14].

The TPA cross-section is an important microcosmic indicator of TPA performance [15]. In order to increase the TPA cross-section, organic molecular design is usually achieved through adding π-conjugated bridges [16], and the complex molecular structure will cause structural distortion but not necessarily increase the TPA cross-section [17]. Therefore, finding simple molecular structures to achieve effective electron delocalization has become a hot topic of research. Recently, a series of derivatives produced through combining organic luminescent materials with 1,3,5-triazine have shown high TPA cross-sections, aggregation-induced emission characteristics, and high quantum efficiency due to electron delocalization interactions among various organic chromophobes [16,18,19]. The substitution or extension of conjugation length by a strong electron donor has a significant influence on TPA/two-photon fluorescence (TPF) and excited-state decay characteristics [20]. Tromayer M. et al. studied the synergistic enhancement effect of the TPA sections of 1,3,5-triazine derivatives containing 1–3 amino-styrenyl donor arms in dipole, quadrupole, and octupole push–pull systems, showing that the molecular dipole moment absorption cross-sections have a significant influence [21]. The distance-dependent and resonance electron-donating effects indicate that fluorine, chlorine, and bromine may exhibit two opposite effects on HOMO/LUMO energy levels and charge distribution within the orbitals. The observed photophysical behavior may be potentially different when the location of bromine on the branch chain and the distance between bromine and phenyl in the biphenyl subunit are different [22]. In order to investigate the mechanism of the molecular dipole moment regulating the TPA cross-section, we studied the OPA and TPA properties of a molecular system of bromobiphenyl combined with 1,3,5 triazine. Finally, we calculated the ECD spectrum of molecular systems to characterize the effect of molecular planarity on molecular chirality.

## 2. Results

### 2.1. OPA

OPA is the process of electron-absorbing photons transitioning from ground state excitation to excited states. Vertical absorption is usually used to study the transition mode of the excited state. It is necessary to optimize the geometric structure of the ground state and calculate the excitation energy from the ground state to the excited state based on the optimized structure.

The physical quantity reflecting the intensity of absorption of a single photon is the oscillator strength, which is determined according to the transition dipole moment and energy difference between the initial and the final states of the electron:

(1) $$f=\frac{2\Delta E^{*}{\left|\langle i\left|-r\right|j\rangle \right|}^{2}}{3}$$

where $|i\rangle$ and $|j\rangle$ is the wave function of the initial state and r is the final state of the electron and is the coordinate vector. The process of obtaining the ultraviolet-visible spectrum (UV-vis spectrum) requires broadening the calculated excitation energy and oscillator strength using a Gaussian function:

(2) $$G(\omega )=\frac{1}{c\sqrt{2\pi }}e^{-\frac{{\left(\omega -\omega_{i}\right)}^{2}}{2c^{2}}}$$

where $c=\frac{FWHM}{2\sqrt{2\ln2}}$, ω is the abscissa of the spectrum. After the transition energy $\omega_{i}$ and FWHM are given, the absorption curve of the transition can be obtained, and the superposition of all the transitions gives the UV-vis spectrum.

### 2.2. TPA

TPA is the process of material absorbing two photons from ground state excitation to the excited state, which belongs to the third-order nonlinear optical effect, and its electron excitation process can be reflected in the following formula [23].

(3) $$\delta_{tp}=8\sum_{\begin{array}{l} j\neq g\\ j\neq f \end{array}}\frac{\left|\langle f\left|\mu \right|\rangle j\right|{\left|\langle j\left|\mu \right|g\rangle \right|}^{2}}{\left(\omega_{j}-\omega_{f}/2\right)+\Gamma_{f}^{2}}\left(1+2\cos^{2}\theta_{j}\right)+8\frac{{\left|\Delta \mu_{fg}\right|}^{2}{\left|f\langle \mu \rangle g\right|}^{2}}{{\left(\omega_{f}/2\right)}^{2}+\Gamma_{f}^{2}}\left(1+2\cos^{2}\phi \right)$$

where f, j, and g are the electron wave functions of the initial state, the intermediate state, and the final state, respectively. $\Gamma_{f}^{2}$ is the lifetime of the final state. $\langle f\left|\mu \right|j\rangle$, $\langle j\left|\mu \right|g\rangle$, and $\langle f\left|\mu \right|g\rangle$ are the transition dipole moments from the ground state to the intermediate state, the intermediate state to the final state, and the transition dipole moments from the ground state to the final state, respectively. $\theta_{j}$ is the angle between the transition dipole moment vectors $\langle j\left|\mu \right|g\rangle$ and $\langle f\left|\mu \right|j\rangle$. $\Delta \mu_{fg}$ is the transition dipole moment between the ground state and the final state, which can be obtained from Formula (4):

(4) $$\Delta \mu_{fg}=\langle f\left|\mu \right|f\rangle -\langle g\left|\mu \right|g\rangle$$

where ϕ is the angle between the transition dipole moment vectors, and $\omega_{i}$ and $\omega_{j}$ are the excitation energies of the intermediate and final states, respectively.

The TPA intensity depends on the absorption cross-section [24], which can be obtained from Formula (5):

(5) $$\sigma^{TPA}=\frac{N\pi^{3}\alpha^{2}\hbar^{3}\omega^{2}}{15e^{4}}\gamma^{TPA}$$

where γTPA=∑αβ(Imγααββ+Imγαββα+Imγαβαβ) is the second-order polarizability in reduced form. $\alpha =\frac{e^{2}}{4\pi \varepsilon_{0}c\hbar }$ is the fine structure constant, e is the electron charge, $\varepsilon_{0}$ is the vacuum dielectric constant, c is the vacuum speed of light, and ħ is the reduced Planck constant. In a simulated TPA spectrum, the integer N is usually taken as 4, and the value of $\sigma_{TPA}$ is usually in units of Göppert Mayer (1 GM = 10^−50^ cm^4^·s·photon^−1^).

### 2.3. Transition Density Matrix (TDM) and Electron Hole Pair Density

For a multi-electron system, the real space form $T(r;r^{\prime })$ between the ground state and the excited state is as follows [25,26]:

(6) $$T(r;r^{\prime })\equiv T(r_{1};{r_{1}}^{\prime })=\int \Phi^{0}(x_{1},x_{1},\cdots x_{N})\Psi^{\mathrm{exc}}({x_{1}}^{\prime },x_{1},\cdots x_{N})d\sigma_{1}dx_{2}dx_{3}\cdots dx_{N}$$

where $\Phi^{0}$ and $\Psi^{\mathrm{exc}}$ represent a ground state wave function and an excited state wave function, x is the spin space coordinate of the electron, σ is the spin space coordinate, and r is the space coordinate. When calculating the excited state using a single reference method such as Time-Dependent Density Functional Theory (TDDFT), the excited state wave function is described using a linear combination of various single excited configuration functions, and $T(r;r^{\prime })$ can be written as:

(7) $$T(r;r^{\prime })=\sum \sum \omega_{i}^{a}\varphi_{i}(r)\varphi_{a}(r^{\prime })$$

where a and i are the numbers of vacant and occupied orbitals, respectively, and ω is the coefficient of the configuration function. Since r contains three components, $T(r;r^{\prime })$ is a six-dimensional function, which cannot be represented simply using images. It is necessary to further take diagonal elements for TDM. $T(r;r^{\prime })$ becomes a three-dimensional function as $r=r^{\prime }$, which can be easily graphed.

Since the concept of transition density is abstract, consider a simple electron transition. The electron excitation can be perfectly described using a transition from an occupied orbital i to an a. It can be considered that where the transition density is higher as $T(r)=\varphi_{i}(r)*\varphi_{a}(r)$, the corresponding orbitals i and a overlap more significantly.

The actual electron excitation cannot be described perfectly using a pair of orbital transitions, but should be broadly described as the transfer from hole to electron. For TDDFT, excited state wave function is described using the linear combination of a single excited configuration function [27]:

(8) $$\rho_{\left(\mathrm{loc}\right)}^{\mathrm{hole}}(r)=\sum_{i\to a}{\left(w_{i}^{a}\right)}^{2}\varphi_{i}\varphi_{i}-\sum_{i\leftarrow a}{\left(w_{i}\prime^{a}\right)}^{2}\varphi_{i}\varphi_{i}$$

(9) $$\rho_{\left(\mathrm{loc}\right)}^{\mathrm{hole}}(r)=\sum_{i\to a}{\left(w_{i}^{a}\right)}^{2}\varphi_{i}\varphi_{i}-\sum_{i\leftarrow a}{\left(w_{i}\prime^{a}\right)}^{2}\varphi_{a}\varphi_{a}$$

In the above equation, r is the coordinate vector, φ is the orbital wave function, i or j is the occupied orbital label, and a or b is the vacant orbital label. $\sum_{i\to a}$ stands for loop every excitation configuration, and $\sum_{i\leftarrow a}$ stands for loop every deexcitation configuration. If an electron excitation can be perfectly described using a pair of orbital transitions, then the contribution of the configuration function is exactly 100%, $\varphi_{i}$ and $\varphi_{a}$ can be used directly and ideally as holes and electrons, respectively. According to this definition, ${\left|\varphi_{i}\right|}^{2}$ and ${\left|\varphi_{a}\right|}^{2}$ can be viewed as the electron densities of the i and a orbitals. The actual excited state of electron involves many configuration functions, so it is necessary to define the electron and hole in detail and take all orbital transitions into account. The hole defined in this way is a good description of the region where excited electrons leave and gather, which can fully display the characteristics of electron excitation.

The ECD spectrum can effectively characterize the chirality of 1,3,5 triazine derivatives, which is the tensor product between the transition electric dipole moment and transition magnetic dipole moment; its intensities are defined as [28]:

(10) $$I\propto \langle \varphi_{j}\left|\mu_{e}\right|\varphi_{i}\rangle \langle \varphi_{j}\left|\mu_{m}\right|\varphi_{i}\rangle B$$

where $\mu_{e}$ and $\mu_{m}$ represent transition electric dipole moment and transition magnetic dipole moment, respectively. The first term represents light absorption and the second represents circular dichroism.

**Figure 1:**
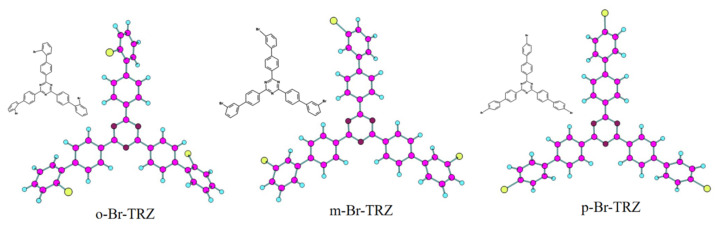
Molecular structure of bromobiphenyl derivatives bound to 1,3,5 triazines.

The constructed molecular model is shown in Figure 1. Triazine is successively combined with 2-bromobiphenyl, 3-bromobiphenyl, and 1-bromobiphenyl to form a three-branched chain molecular structure (o-Br-TRZ, m-Br-TRZ, and p-Br-TRZ). When the photoactive groups Br and triazine interact with ultraviolet light (UV-light), the three-branched chain of bromobiphenyl acts as a rotor to drive molecular motion and produce a unique photothermal effect [23].

**Figure 2:**
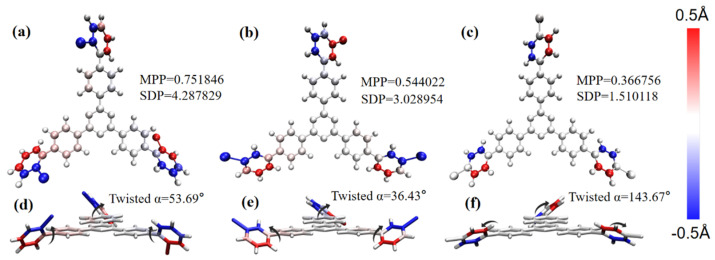
(**a**–**c**) Coloring diagram of MPP and atomic SDP of 1,3,5 triazine derivatives of bromobiphenyl as a branched chain; (**d**–**f**) schematic diagram of dihedral angle on bromobiphenyl branch chain.

The optimized molecular structure is shown in Figure 2. In order to investigate the influence of the change in the key point between Br and the benzene ring in the molecular structure, we calculated the planarity of the three structures and drew the coloring diagram of each atom deviating from the fitting plane. The more the atom deviates from the fitting plane, the darker the coloring will be. Blue and red respectively represent the atoms below and above the fitting plane. The two methods of characterizing planarity are the molecular planarity parameter (MPP) and span of deviation from plane (SDP), which are obtained using the following formula:

(11) $$MPP=\sqrt{\frac{1}{N_{atom}}\sum_{i}d_{i}^{2}}$$

(12) $$SDP=d_{\max}^{s}-d_{\min}^{s}$$

where $N_{atom}$ is the total number of atoms considered. $d_{i}^{2}$ represents the distance of the i atom from the fitting plane, and $d_{\max}^{s}$ and $d_{\min}^{s}$ are, respectively, the most positive and negative values of d_s in all the atoms considered.

The dihedral angles of o-Br-TRZ, m-Br-TRZ, and p-Br-TRZ branches to the fitted plane are 53.69°, 36.43°, and 143.67°. The MPP and SDP values of o-Br-TRZ are the largest, indicating that the overall flatness, the span perpendicular to the fitting plane, and the degree of atomic deviation from the fitting plane are the largest. p-Br-TRZ has the largest dihedral angle, but the flatness is the weakest, as shown in Figure 2a–c. It can be seen that with the increasing bonding position of Br and the benzene ring, the twisted chain inside the branch of bromobiphenyl becomes smaller, and the planar property becomes weaker. This torsion will change the dipole moment of the molecule and affect its optical properties.

## 3. Discussion

### 3.1. OPA Spectrum and TPA Spectrum

**Figure 3:**
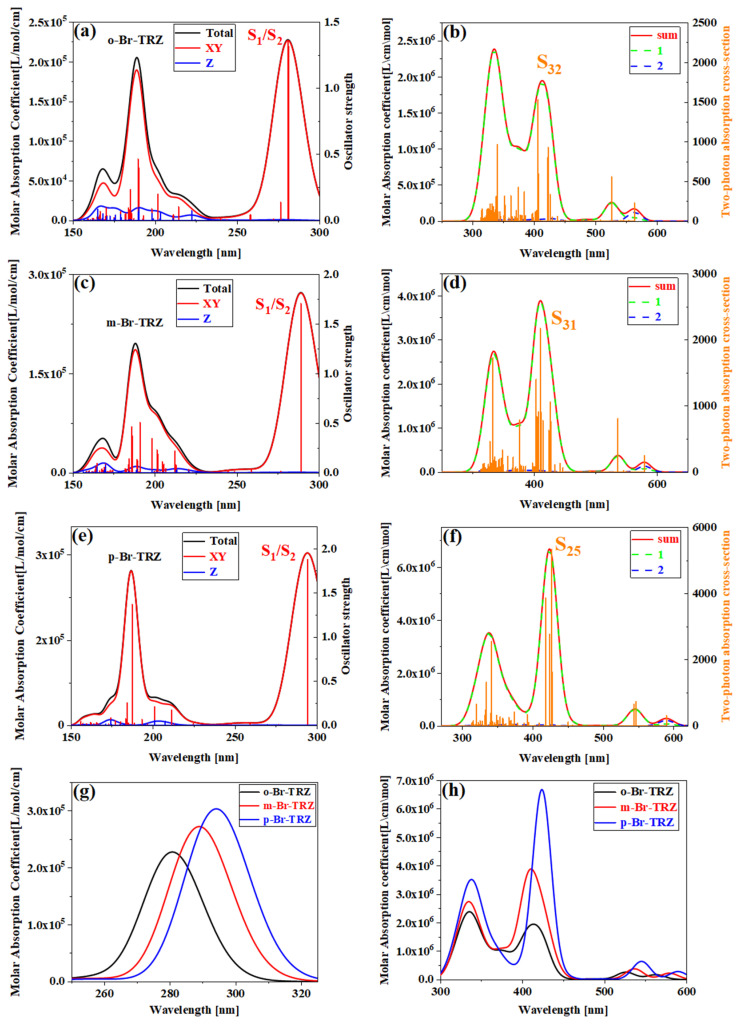
(**a**,**c**,**e**) OPA spectrum of o-Br-TRZ, m-Br-TRZ, and p-Br-TRZ; (**b**,**d**,**f**) TPA spectrum and their combined graphs (**g**,**h**).

In order to analyze the effect of planarity on the optical absorption characteristics of triazine derivatives, the interaction between the oscillating electric field corresponding to electromagnetic waves in different incident directions and the system should be considered first. For systems with strong molecular planarity, it is useful to examine separately the contributions of the different Cartesian components of the UV-vis spectrum, which allows us to more fully understand the optical absorption of different wavelengths from the interaction between the system and the oscillating electric field and in what direction. We calculated the UV-vis spectrum in the XY direction (red line) and Z direction (blue line), as shown in Figure 3. It can be seen that the main absorption peak at 250–300 nm is contributed by two degenerate excited states S_1_ and S_2_ together. The strongest absorption peaks of o-Br-TRZ and m-Br-TRZ come from the interaction between the oscillating electric field and the system in the XY direction. Only in the extreme ultraviolet wavelength region, the interaction between the oscillating electric field in the Z direction and the system contribute, as shown in Figure 3a,c. For p-Br-TRZ, the contribution of the oscillating electric field in the Z direction to the interaction with the system is small, because the structure is the weakest in the plane line at a large twisted angle, as shown in Figure 3e. With the constant increase in twisted angle in the bromobiphenyl unit, a redshift occurred in the system, as shown in Figure 3g.

The extreme ultraviolet absorption limited the application of triazine derivatives. In order to broaden the absorption range, the TPA spectra of triazine derivatives were calculated. In accordance with the OPA spectrum, TPA is mainly composed of large cross-section absorption at 300–500 nm and small cross-section absorption at 500–600 nm. The TPA consists of two transition mechanisms, including the first term transition from the ground state to the intermediate state and the second transition from the intermediate state to the final state, as well as the second term transition from the ground state to the final state. The green dotted line represents the first term, the blue dotted line represents the second term, and the black line represents the sum of the two step transition terms. It can be clearly seen that the strongest absorption peaks of o-Br-TRZ, m-Br-TRZ, and p-Br-TRZ are contributed by S_32_, S_31_, and S_25_, respectively. The red and green curves of the absorption peaks have a high fitting degree, indicating that the two-step transition dominates in TPA, and the maximum TPA cross-section of TPA spectrum increases by 3.5 times from 1500 GM to 5000 GM, as shown in Figure 3b,d,f. From the sum of the first and second terms, the TPA spectrum has an expected redshift within the visible light absorption range, as shown in Figure 3h.

### 3.2. Electron Excitation Characteristics of OPA

**Figure 4:**
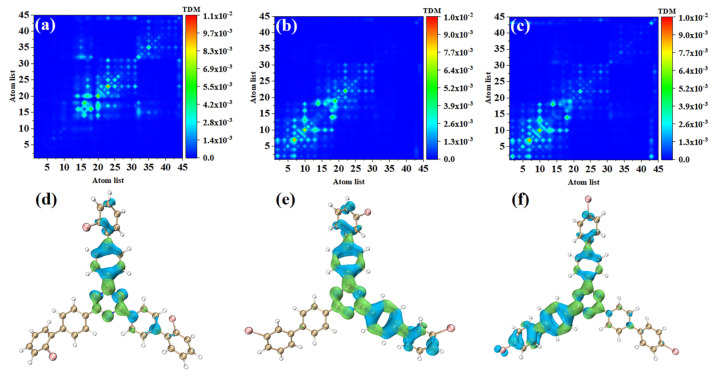
(**a**–**c**) TDM and (**d**–**f**) electron hole pair densities of o-Br-TRZ, m-Br-TRZ, and p-Br-TRZ at S_1_.

In order to study the physical mechanisms of o-Br-TRZ, m-Br-TRZ, and p-Br-TRZ in the redshift of the UV-vis spectrum, it is necessary to analyze the electronic excitation characteristics during the interaction between light and physics. We plotted the TDM and electron hole pair density corresponding to the strongest absorption peaks of the three structures, and we can effectively discuss the CT and local excitation state (LE) using the process of electron transition, which is superior to the molecular orbital (MO) analysis method (see Table S1 in Supplementary Materials). Bright areas in TDM are regions with higher transition density, corresponding to places with large electron hole overlap, and the abscis and ordinates are atomic numbers. Figure 4a shows that the transition density is mainly concentrated on the main diagonal line, indicating that electron excitation mainly occurs between adjacent atoms and the LE is dominant within the molecule. In order to investigate the spatial distribution characteristics of electron transition, it is necessary to combine the electron hole pair density. In Figure 4d, the blue isosurface represents the area of electron concentration, and the green isosurface shows the area of holes left after the electrons leave. It can be seen that the electron hole pair density mainly concentrates on the bromobiphenyl branch chain above. A few electrons are distributed in the right branch chain, and no electron transition occurs in the left branch chain. The direction of charge transfer is from the central benzene ring of o-Br-TRZ to the bromobiphenyl branch chain, and in the middle of the bromobiphenyl branch chain can be seen the obvious electron hole overlap region.

The electron excitation characteristics of m-Br-TRZ and p-Br-TRZ are similar, both of which are LE, and o-Br-TRZ has a larger isosurface distribution, as shown in Figure 4b,c. From the perspective of electron hole pair density, this is mainly because m-Br-TRZ and p-Br-TRZ have significant electron hole pair density on one bromobiphenyl branch chain (corresponding to the right branch chain and the left branch chain, respectively), but also on the upper branch chain.

**Figure 5:**
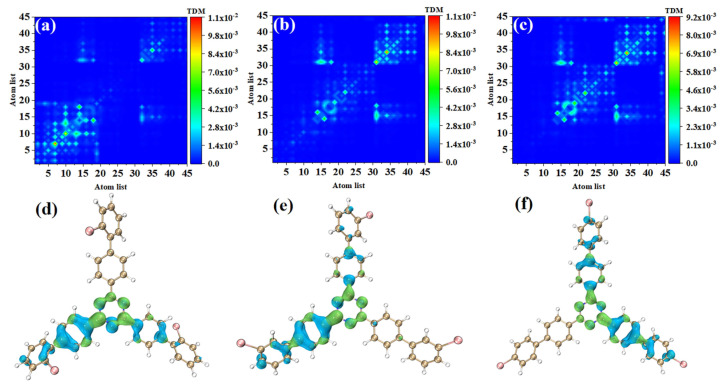
(**a**–**c**) TDM and (**d**–**f**) electron hole pair densities of o-Br-TRZ, m-Br-TRZ, and p-Br-TRZ in S_2_.

S_2_ and S_1_ are degenerate excited states of the main absorption peak, and the oscillator strength are very close; thus, the corresponding electron transition contribution to the main absorption peak of the UV-vis spectrum is similar. The TDM of o-Br-TRZ in S_2_ is similar to S_1_, both of which belong to LE states, see Figure 4d. The difference is that the main region of electron hole distribution changes from the upper side chain of bromobiphenyl to the left side chain, and the electron hole distribution on the upper branch chain almost completely dissipates. The electron hole distribution of the two excited states happens to be very complementary, as shown in Figure 5d. The TDM of o-Br-TRZ shows that S_2_ is also an LE state. The difference is that the transition density is also partially distributed on both sides of the diagonal, indicating that a small amount of CT occurs within the molecule, as shown in Figure 5b. The region of electron hole distribution changes from the right branch chain to the left branch chain, and the region of electron hole distribution of S_1_ and S_2_ is also complementary, as shown in Figure 5e. The electron transition properties of p-Br-TRZ in S_2_ are similar to those of o-Br-TRZ and m-Br-TRZ.

We have studied the electron transition characteristics of molecular systems at 250–300 nm light absorption from a qualitative point of view. In order to deeply reveal the physical mechanism of the redshift in the absorption peak, it is also necessary to quantitatively investigate electron hole transition characteristics. Table 1 shows the transition index obtained from the wave function analysis of the electronic excited state. We first define the $D_{index}$ of the distance between the holes and the electronic center of mass:

(13) $$D_{\mathrm{index}}=\sqrt{{\left(D_{x}\right)}^{2}+{\left(D_{y}\right)}^{2}+{\left(D_{z}\right)}^{2}}$$

where $D_{x}=\left|X_{ele}-X_{hole}\right|$, $D_{x}=\left|Y_{ele}-Y_{hole}\right|$, and $D_{x}=\left|Z_{ele}-Z_{hole}\right|$. It can be seen that the distance between the hole and the electron centroid of all excited states is about 0.78–1.0 Å; only p-Br-TRZ has a greater $D_{index}$ value more than 1 in S_2_. From the perspective of electron hole pair density, this is due to the uniform distribution of hole–electron isosurface on the upper and right branches. $S_{r}$ is a function describing the overlap between electron and hole distribution, and its range is 0 to 1; larger values represent a greater degree of electron hole overlap, which is obtained from the formula $S_{r}(r)=\sqrt{\rho^{\mathrm{hole}}(r)\rho^{\mathrm{ele}}(r)}$, where $\rho^{hole}(r)$ and $\rho^{ele}(r)$ are spatial distribution functions of hole electrons, respectively. The $S_{r}$ of o-Br-TRZ in S_1_ is larger than the others, indicating that the degree of electron hole overlap is large. The physical opposite of the degree of overlap of electron holes is $t_{index}$, which measures the degree of separation of electron and hole:

(14) $$t_{\mathrm{index}}=D_{index}-H_{CT}$$

where $H_{CT}$ represents the average degree of hole and electron extension in the CT direction. It can be seen all the $t_{index}<0$ in excited states, which indicates that there is no significant separation between hole and electron in the direction of CT, which accords well with the previous conclusion that electron excitation in molecular systems belongs to LE. Considering that electron holes transfer from one bromobiphenyl branch chain to another bromobiphenyl branch chain at different positions of Br, the spatial distribution characteristics change significantly, and it is also necessary to investigate the overall average distribution span $H_{index}$ and the difference of the overall spatial distribution span Δσ, which can be obtained using the following formulas:

(15) $$H_{index}=\left(\left|\sigma_{ele}\right|+\left|\sigma_{hole}\right|\right)/2$$

(16) $$\Delta \sigma_{index}=\left|\sigma_{ele}\right|-\left|\sigma_{hole}\right|$$

where $\sigma_{ele}$ and $\sigma_{hole}$ are the overall spatial distribution breadth of electrons and holes, respectively. It can be seen that m-Br-TRZ has a smaller $H_{index}$, while o-Br-TRZ and p-Br-TRZ are very close. p-Br-TRZ has the largest Δσ in S_1_ and S_2_, which indicates that the spatial distribution breadth of the holes in these two excited states is larger than the electrons. It can be found from quantitative analysis that the electron hole characteristics of triazine derivative molecules in the process of electron transition will be significantly different with the change of bonding position between Br and the benzene ring. Under different wavelength excitation, the LE on the bromobiphenyl branch chain will be transferred from one branch chain to another, and the decrease of electron excitation energy will result in the redshift of the UV-vis spectrum. In addition, the interfragment charge transfer (IFCT) can assist in the analysis of the contribution of charge transfer between fragments to the excited state, which subjectively shows the contribution of charge transfer between segments to electrons and holes (see Figures S1–S3 in Supplementary Materials).

### 3.3. Electron Excitation Characteristics of TPA

**Figure 6:**
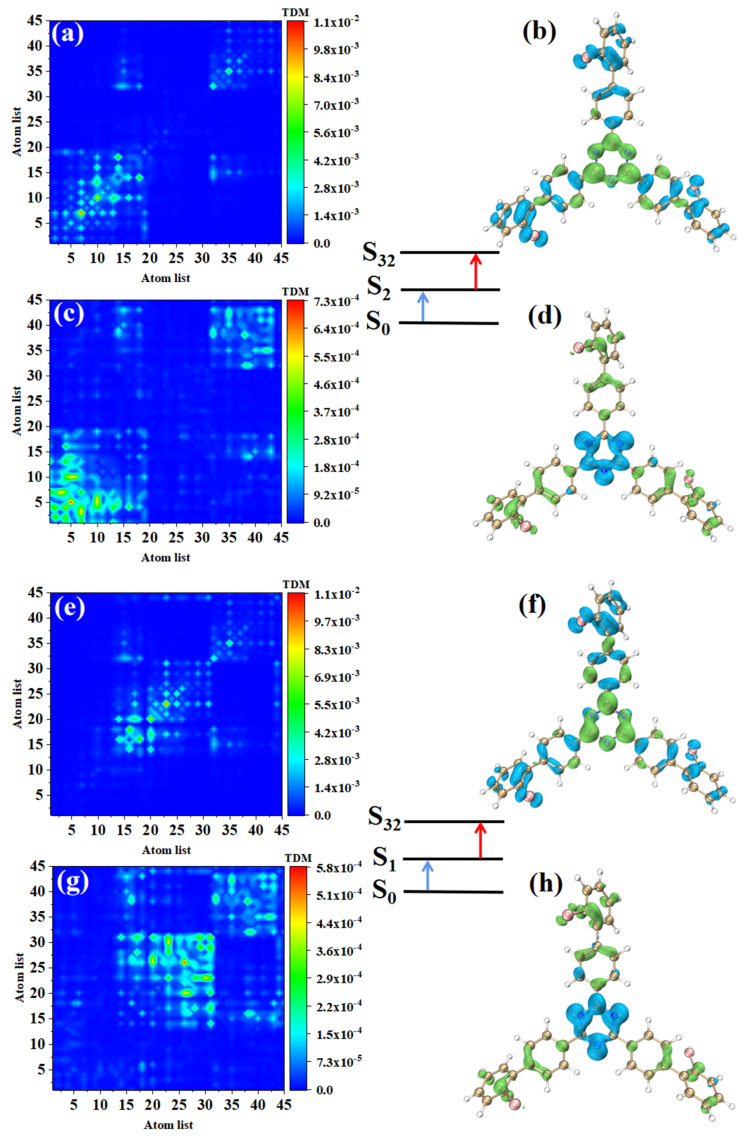
The two-step transition process of o-Br-TRZ in S_32_, the CT characteristics of the (**a**,**b**) first step transition and the (**c**,**d**) second step transition for intermediate state S_2_, and the (**g**,**h**) first step transition and (**e**,**f**) second step transition for intermediate state S_1_ (the blue isosurface of the electron hole pair density represents the electron and the green isosurface represents the hole).

The main index reflecting the absorption intensity of two photons is the absorption cross-section. Table 2 shows the two-step transition process of TPA excited states with the strongest absorption peaks of the three molecules. The electron transition of these excited states generates the absorption cross-sections from the electronic transitions to these excited states. It can be seen that the absorption cross-sections increase 3.5 times with the increase of the permanent dipole moment of the two-step transition. In order to analyze the physical mechanism of the increase in the TPA cross-section, it is necessary to analyze the process of two transitions, which is a one-photon excitation and a transition from an excited state to another excited state. We drew the electron hole pair density and TDM of each step to discuss the excited state characteristics of this two-transition process. Table 2 shows that the TPA of o-Br-TRZ in S_32_ includes two transition paths. The intermediate state of the first path is S_2_, and its first step transition, S_0_→S_2,_ is the transition from the ground state S_0_ to S_2_. The electron excitation characteristics are the same as those of S_2_ in OPA, both of which belong to the LE (see Figure 6a). The electron hole pair density shows the transfer of electrons from triazine to the bromobiphenyl branch chain under photoexcitation, resulting in hole regions distributed around the six-membered ring of triazine, as shown in Figure 6b. The second step is the transition from the intermediate state to final state, S_2_→S_32_. From the perspective of TDM, the transition density is mainly distributed on both sides of the main diagonal, and some bright areas near the main diagonal are significant, so S_2_→S_32_ belongs to CT, as shown in Figure 6c. According to the electron hole pair density, electrons transfer from the bromobiphenyl branch chain to triazine, forming a significant electron aggregation region around the six-membered ring of triazine, and the degree of electron hole separation is quite sufficient, as shown in Figure 6d. The intermediate state of the second path is S_1_, and the first step transition S_0_→S_2_ belongs to LE. It can be seen that electrons and holes are not completely separated in the three bromobiphenyl branch chains, and there is a large degree of overlap (see Figure 6e,f). The second transition S_1_→S_32_ belongs to charge transfer excitation and differs from S_2_ in that the distribution region of transition density is different. From the perspective of electron hole pair density, this is because the electron cross-section appears in the region connecting triazine and the two bromobiphenyl branch chains on the lower side, while the electron cross-section of S_2_→S_32_ appears in the region connecting triazine and the bromobiphenyl branch chains on the upper side.

Combining the two paths, it can be found that the electron excitation changes from LE to CT during the transition from the first step to the second step. The LE of the first step transition is the process of charge accumulation, and the CT of the second step transition is the transfer of accumulated charge excited by the localization of the first step. In the whole electron transition process of TPA, the electron is first partially transferred from triazine to bromobiphenyl branch chain, and then from the bromobiphenyl branch chain to triazine. This transfer can be regarded as a charge sequence transfer.

**Figure 7:**
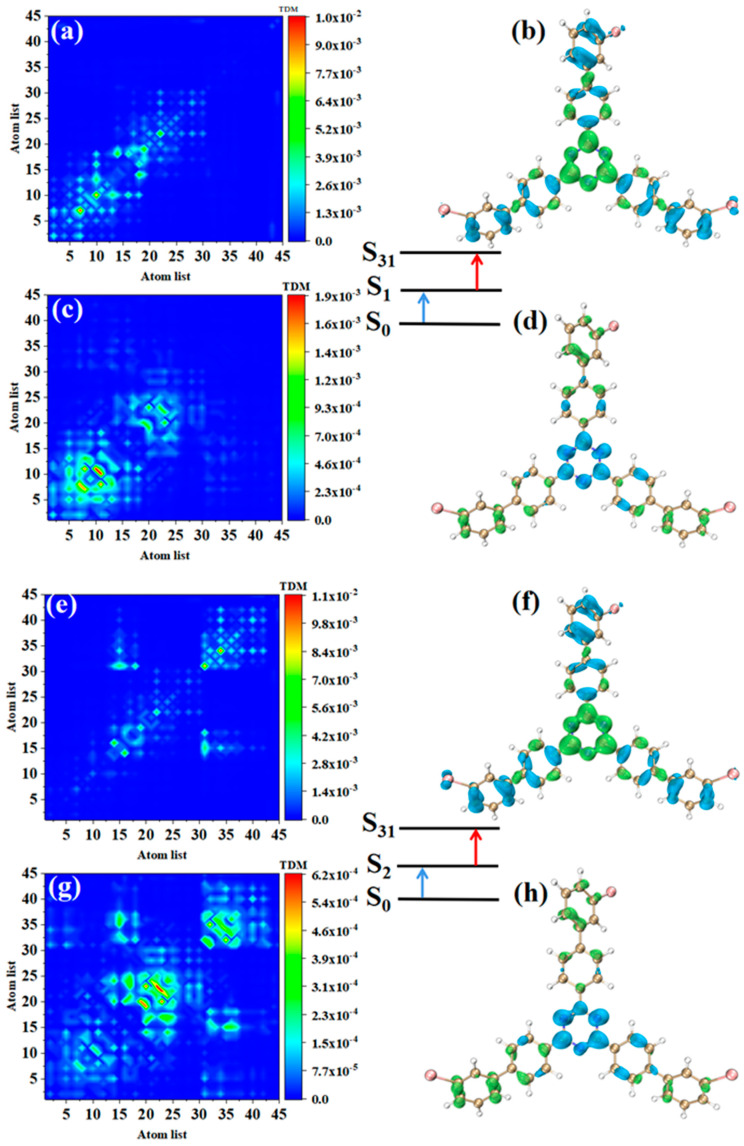
The two-step transition process of m-Br-TRZ in S_31_, the CT characteristics of the (**a**,**b**) first step transition and the (**c**,**d**) second step transition for intermediate state S_2_ and the (**g**,**h**) first step transition and (**e**,**f**) second step transition for intermediate state S_1_ (the blue isosurface of the electron hole pair density represents the electron and the green isosurface represents the hole).

The TPA of m-Br-TRZ also includes two paths. When the intermediate state is S_1_, the first step transition S_0_→S_1_ is similar to o-Br-TRZ (refer to Figure 7a,b). The second step transition S_1_→S_31_ belongs to CT, and TDM shows that the transition density is closer to the main diagonal, indicating that the intracolecular LE is enhanced. A small amount of electron hole overlap can be seen in the connection region of bromobiphenyl and triazine, and it can be clearly observed that the electron isosurface on the six atoms of triazine appear in cross-section, as shown in Figure 7c,d. 

**Figure 8:**
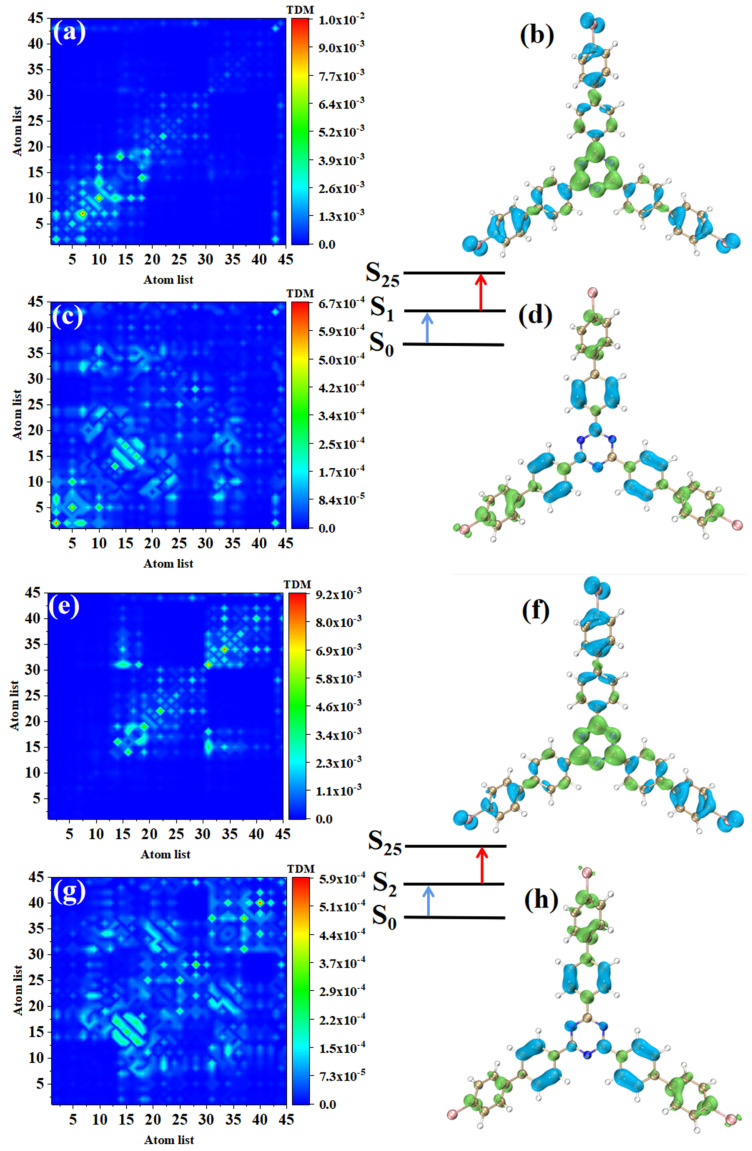
The two-step transition process of p-Br-TRZ in S_25_, the CT characteristics of the (**a**,**b**) first step transition and the (**c**,**d**) second step transition for intermediate state S_2_, and the (**g**,**h**) first step transition and (**e**,**f**) second step transition for intermediate state S_1_ (the blue isosurface of the electron hole pair density represents the electron and the green isosurface represents the hole).

The electronic transition characteristics of the intermediate state S_2_ are similar to those of the intermediate state S_1_, as shown in Figure 7e–h. The TPA of p-Br-TRZ consists of two transition paths with intermediate states S_1_ and S_2_. The first transitions S_0_→S_1_ and S_0_→S_2_ of both paths belong to LE (see Figure 8a,e). The difference is that the TDM of the second step transition shows that the transition density distribution of S_1_→S_31_ and S_2_→S_31_ on both sides of bromobiphenyl becomes wider, but the brightness decreases significantly. The bright area is mainly concentrated in the region near the main diagonal, as shown in Figure 8c,g. Therefore, the LE characteristics of the second step transition are enhanced, and the CT characteristics are weakened. As shown in electron hole pair density, electrons are no longer concentrated around the six-membered ring of triazine but diffuse to the first benzene ring connected to bromobiphenyl, as shown in Figure 8d,h.

The first step of TPA in triazine derivatives is LE. Under the action of two photons, the accumulated charge of the first LE will promote the CT excitation of the second step transition, so the spatial distribution of electrons and holes in the two-step transition is exactly symmetric. Generally, the TPA cross-section of CT is weaker; as the CT of the second step transition becomes weaker, p-Br-TRZ obtains a larger TPA cross-section, which is nearly 3.5 times that of o-Br-TRZ.

### 3.4. Transition Dipole Moment Analysis of Molecular Fragments

**Figure 9:**
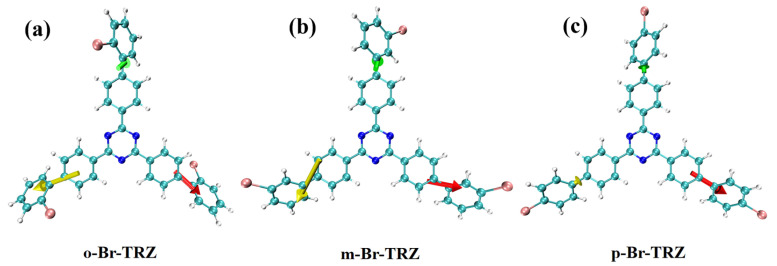
(**a**–**c**) The transition dipole moment of molecular fragment of bromobiphenyl branch in S_32_, S_31_, and S_25_ of o-Br-TRZ, m-Br-TRZ, and p-Br-TRZ, respectively (green, yellow, and red arrows represent molecular dipole moments on three different branch chains, length represents size, and arrows represent vector direction).

The CT in molecular systems is induced by the permanent dipole moment. In order to analyze the physical mechanism of weakened CT in TPA, we drew the dipole moment of the molecular fragment diagrams of o-Br-TRZ, m-Br-TRZ, and p-Br-TRZ at S_32_, S_31,_ and S_25_. The transition dipole moments of the three bromobiphenyl branch chains are represented with vector arrows in three different colors. The direction of the arrows represents the direction of the transition dipole moment vector, and the length of the arrows represents the dipole moment magnitude. It can be seen that the direction of the fragment transition dipole moment on the left branch chain of m-Br-TRZ changes significantly with the change of the benzene ring bonding position in Br, as shown in Figure 9a,b. When the key point of Br and the benzene ring is at the outer end, the transition dipole moment on the left branch chain is opposite to o-Br-TRZ and m-Br-TRZ, and the cylindrical part representing the value of the transition dipole moment becomes weak (see Figure 9c). This greatly weakens the permanent dipole moment of the molecular structure, resulting in a lower degree of charge transfer and a larger absorption cross-section under TPA.

### 3.5. Electrostatic Potential Analysis of Molecular Surface

**Figure 10:**
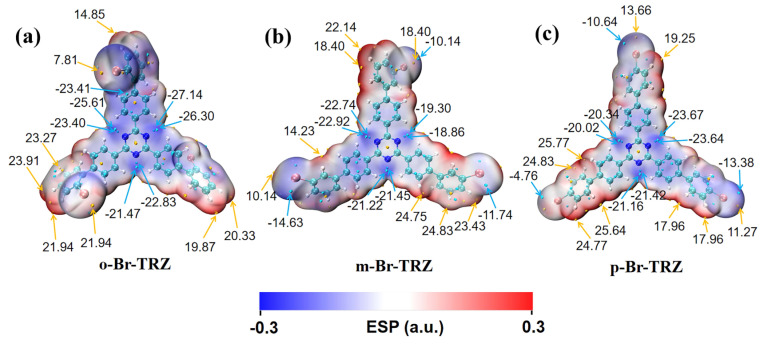
(**a**–**c**) Molecular surface electrostatic potential of o-Br-TRZ, m-Br-TRZ, and p-Br-TRZ. (The blue isosurface represents the region with negative potential value, and the red isosurface represents the region with positive potential value. The yellow ball represents the maximum electrostatic potential point, the blue ball represents the minimum electrostatic potential point, and the unit is kcal/mol.).

The molecular dipole moment can be analyzed using the electrostatic potential of the molecular surface, so we draw the electrostatic potential diagram of the molecular surface of three molecular structures. It can be clearly seen that the surface electrostatic potentials of the top molecules of the three branches of bromobiphenyl are very different due to the different bonding of Br. The negative electrostatic potential values were mainly concentrated around the N of the central triazine. The electrostatic potential on the molecular surface of the outer part of the bromobiphenyl branch chain of o-Br-TRZ had an obvious deflection from the internal part, and there was a minimum electrostatic potential of −23.41 kcal/mol near the included angle, as shown in Figure 10a. This is because the bonding position between Br and the benzene ring is close to the interior of the molecule, and the electrostatic interaction is strong. As the bonding position between Br and the benzene ring becomes farther, the deviation degree of the outer part of the bromobiphenyl branch chain decreases. The minimum extreme value of electrostatic potential on the bromobiphenyl branch chain on the left side of m-Br-TRZ is −14.63 kcal/mol, as shown in Figure 10b. The negative electrostatic potential value on the left branch chain of p-Br-TRZ is only −4.76 kcal/mol. On the whole, the range from negative electrostatic potential to positive electrostatic potential is greatly reduced, which will lead to the reduction of kinetic energy obtained in the process of electron transition from high potential to low potential, resulting in the weakening of CT.

### 3.6. Electronic Circular Dichroism Spectra and Chiral Electromagnetic Interaction

**Figure 11:**
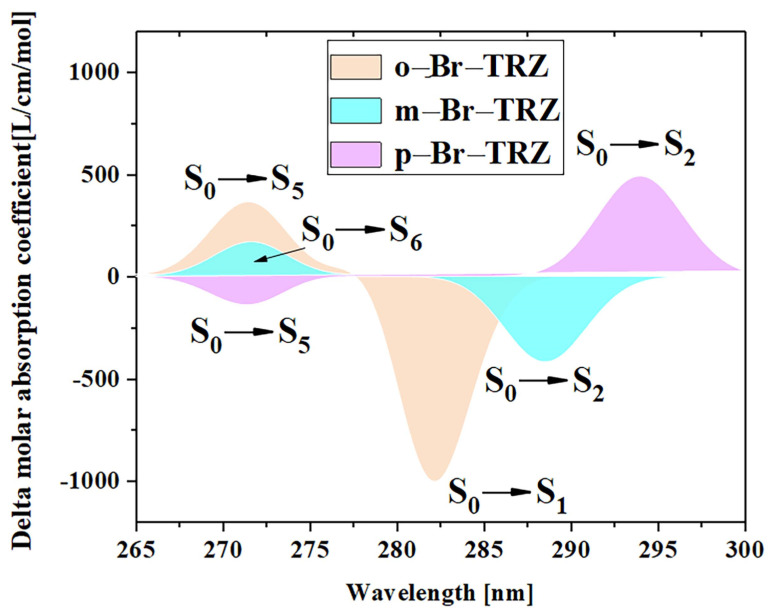
ECD spectrum of three 1,3,5 triazine derivatives.

The different twisted angles of the bromobiphenyl branch chains of 1,3,5 triazine derivatives result in the breaking of molecular symmetry, resulting in chirality. The ECD spectrum can be used to characterize the chirality of chromophones in organic molecules. In order to study the regulatory mechanism of molecular planar chiral properties, the ECD spectrum of three 1,3,5 triazine derivatives were plotted, as shown in Figure 11. It can be seen that the ECD spectrum from 265 nm to 300 nm contains two absorption peaks. The first absorption peak of the three 1,3,5 triazine derivatives is contributed by S_1_, S_2_ and S_3_, respectively, and has the characteristics of redshift, which is mainly caused by the decrease of electron excitation energy. In the ECD spectrum, the intensity of interaction between the molecular system and the electromagnetic wave is characterized by the rotational strength. The first absorption peak of o-Br-TRZ is obtained through broadening the rotational strength of the two excited states, in which the negative rotational strength of excited state S_1_ contributes the main absorption. The first absorption peak of m-Br-TRZ is also obtained through broadening the rotational strength of the two excited states, in which the negative rotational strength of S_2_ plays a leading role. However, the absolute value of the rotational strength decreased, and as the deviation of bromobiphenyl branch chains from the molecular plane was further weakened, the positive rotational strength of the two excited states in p-Br-TRZ dominated, and the corresponding ECD spectrum was positive, as shown in Figure S4. The ECD spectrum of the second absorption peak of the three molecules was contributed by S_5_, S_6,_ and S_5_, respectively. The ECD spectrum changed from positive to negative, and the chirality also reversed. The difference is that the spectrum has no redshift.

**Figure 12:**
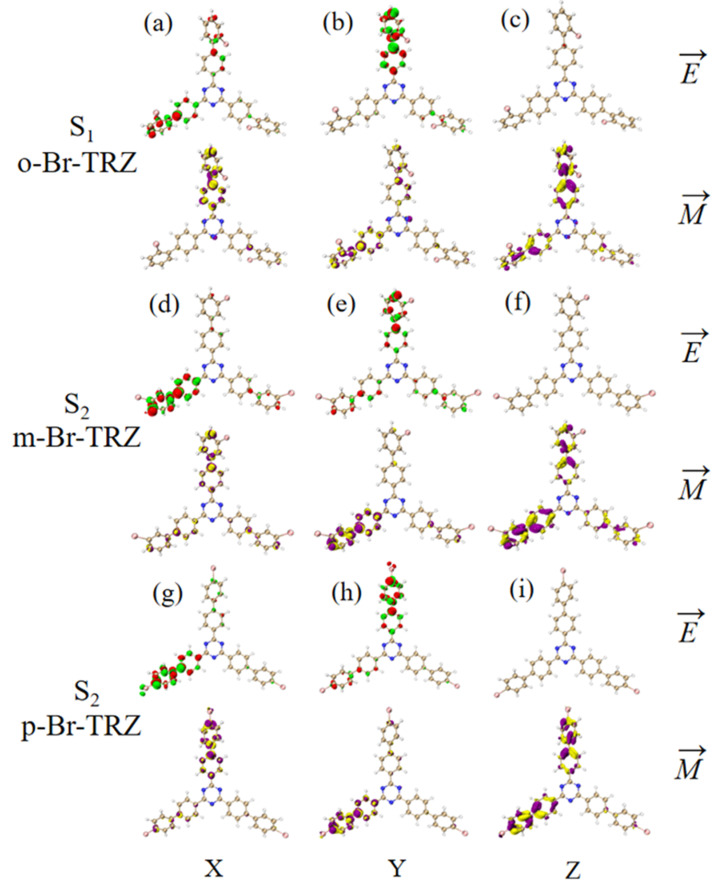
The Cartesian component of TEDM and TMDM in S_1_ of o-Br-TRZ (**a**–**c**); the Cartesian component of TEDM and TMDM in S_2_ of m-Br-TRZ (**d**–**f**); the Cartesian component of TEDM and TMDM in S_2_ of p-Br-TRZ (**g**–**i**).

The ECD is the asymmetric response of the magnetic transition dipole moment and the electric transition dipole moment when the system is interacting with an electromagnetic wave. In order to study the physical mechanism of chiral inversion caused by molecular planarity, the transition electric dipole moment density (TEDM) and transition magnetic dipole moment density (TMDM) are plotted (the isovalue is 0.05, whereas the green and red isosurface represent the positive and negative values of TEDM, respectively, and the purple and yellow isosurface represent the positive and negative values of TMDM, respectively, and the positive and negative values are related to the direction of the transition electric/magnetic dipole moment). It can be seen that the transition electric dipole moment of o-Br-TRZ at S_1_ is mainly contributed by TEDM in the x and y components, and no TEDM in the z component. The transition magnetic dipole moment is distributed in three components, mainly dominated by the z component (see Figure 12a–c). The TEDM of p-Br-TRZ in the x and y components is similar to that of o-Br-TRZ in the x component, but the TEDM of o-Br-TRZ in x component is also distributed in the upper branch chain, which has a larger distribution breadth. In the y component, the TEDM distribution breadth of p-Br-TRZ is larger, and the difference is that the TMDM of m-Br-TRZ in the z component spreads to the right branch chain, as shown in Figure 12d–f, but this difference is not enough to cause a chiral reversal. When the planarity of the molecule is further enhanced, the positive and negative TEDM isosurface in the z component alternate except for the difference in the spatial distribution breadth of p-Br-TRZ. This alternating change in the direction of the magnetic dipole moment will significantly change the induction of chiral optical activity by the magnetic dipole moment, resulting in chiral inversion.

**Figure 13:**
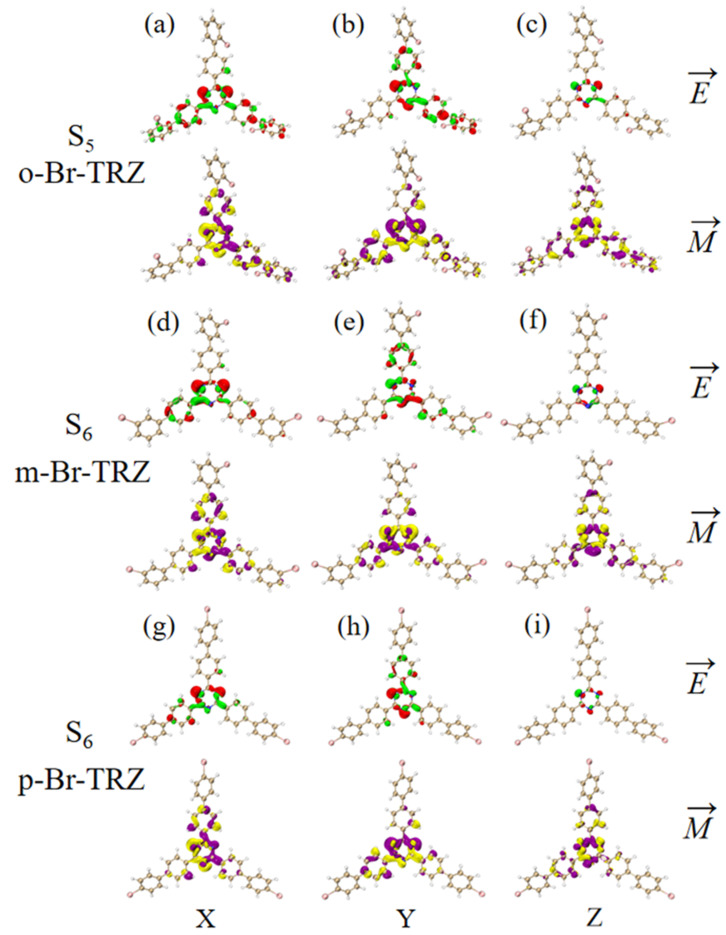
The Cartesian component of TEDM and TMDM in S_5_ of o-Br-TRZ (**a**–**c**); the Cartesian component of TEDM and TMDM in S_6_ of m-Br-TRZ (**d**–**f**); the Cartesian component of TEDM and TMDM in S_6_ of p-Br-TRZ (**g**–**i**).

Since the rotational strength of the second ECD absorption peak is very small, the TEDM and TMDM of the molecular system must be smaller. For the sake of convenient analysis, the value of the isosurface is 0.005. It can be seen that the TEDM of o-Br-TRZ in S_1_ is mainly distributed in the x and y components, and its spatial distribution characteristics decrease from the triazine core to the bromobiphenyl branch chain. The TMDM has a large distribution in all three components, as shown in Figure 13a,b. Compared with o-Br-TRZ, TEDM and TMDM in the three components of m-Br-TRZ decreased, rotational strength decreased, and spectral absorption of ECD decreased (see Figure 13d–f). The TEDM and TMDM distributions of p-Br-TRZ in the x and z components are similar to those of m-Br-TRZ, but the TMDM in the z component has alternating positive and negative values. Therefore, the chiral transition of the second ECD peak comes from the induction of chiral optical activity via the transition magnetic couple of the y component.

## 4. Calculation Details

All quantum chemistry calculations were completed in Gaussian 16 software [27]. The geometric optimization of molecular structures is based on density functional theory (DFT) using a hybrid method composed of Becke’s three-parameter exchange function and Lee–Yang–Parr correlation (B3LYP) functional [29], considering the dispersion of DFT-D3. The basis sets use 6-31G(d) [30], containing the orbital polarization function, and frequency analysis was carried out under functional and basis groups of the same level. After geometric optimization, electronic excited states were calculated using TDDFT [31] to study the vertical excitation energy, oscillator strength, and electronic structural characteristics of excited states. The range separation CAM-B3LYP functional [32] and the 6-311G** basis sets were used, and the dispersion function was used to investigate the weak interaction between Br and the molecular system on the branch chain of bromobiphenyl. The visualization and excitation properties of the OPA, TPA, and ECD spectra were calculated via combining them with the configuration coefficient [33]. TPA cross-section and dipole moment integral analysis were carried out using the method of a self-developed program. The transition information between excited states was calculated via the summation of states (SOS) method based on perturbation theory [34]. The electronic transition analysis of excited states was calculated using the wave function analysis software Multiwfn 3.8 [35], and the visualization of electron excitation in molecular systems was realized in the VMD program [36].

## 5. Conclusions

In this paper, the effects of molecular planarity on the optical absorption characteristics of the 1,3,5 triazine multichain derivatives o-Br-TRZ, m-Br-TRZ, and p-Br-TRZ containing three bromobiphenyl units are investigated theoretically using first principles and wave function analysis. OPA analysis shows that the redshift of the UV-vis spectrum is due to the LE of bromobiphenyl branch chains caused by the decrease of molecular planarity. The spatial distribution of electron holes shifts from one branch chain to another, and the excitation energy of OPA excited states decreases. The molecular dipole moment showed that after the molecular planarity of the multichain derivative was weakened, the molecular dipole moment on the branch chain of bromobiphenyl became smaller and reversed direction. The electrostatic potential analysis shows that with the decrease of planarity, the electrostatic potential difference of triazine derivatives decreases and the intramolecular electrostatic interaction weakens, which weakens the second-step CT of the TPA excited state. The above analysis indicated that the planarity of triazine derivatives could regulate the dipole moment and the electrostatic potential of the molecule and play a role in regulating the TPA cross-section. Molecular chiral analysis indicates that molecular planarity can regulate chiral optical activity through changing the direction of the transition magnetic dipole moment. This study revealed the physical mechanism of molecular planarity regulation of the optical absorption properties of 1,3,5 triazine derivatives, which can provide a theoretical basis for searching for TPA and chiral optical materials with excellent properties from the perspective of molecular structure-absorption properties.

## Figures and Tables

**Table 1:** Transition index of major excited states in OPA of triazine derivatives.

Molecule	Excited States	Excited Energy (eV)	$D_{index}$	$S_{r}$	$t_{index}$	$H_{index}$	Δσ
o-Br-TRZ	S_1_	4.408	0.783	0.877	−2.334	5.549	−0.578
	S_2_	4.415	0.861	0.791	−1.907	4.624	−0.605
m-Br-TRZ	S_1_	4.288	0.913	0.796	−1.535	4.902	−0.597
	S_2_	4.293	0.866	0.798	−2.834	4.911	−0.641
p-Br-TRZ	S_1_	4.215	0.998	0.795	−2.320	5.181	−0.879
	S_2_	4.216	1.003	0.793	−2.651	5.195	−0.890

**Table 2:** The main TPA process and its transition dipole moment value.

Molecule	TPA States	Path	Transition Process	Integral Value (Debye)	TPA Cross-Section (GM)
o-Br-TRZ	S_32_	S_2_	$\langle \phi_{s0}\left|\mu \right|\phi_{s2}\rangle \times \langle \phi_{s2}\left|\mu \right|\phi_{s32}\rangle$	12.592 × 5.180	1.533 × 10^3^
		S_1_	$\langle \phi_{s0}\left|\mu \right|\phi_{s1}\rangle \times \langle \phi_{s1}\left|\mu \right|\phi_{s32}\rangle$	12.213 × 4.223	
m-Br-TRZ	S_31_	S_1_	$\langle \phi_{s0}\left|\mu \right|\phi_{s1}\rangle \times \langle \phi_{s1}\left|\mu \right|\phi_{s31}\rangle$	15.740 × 5.963	2.173 × 10^3^
		S_2_	$\langle \phi_{s0}\left|\mu \right|\phi_{s2}\rangle \times \langle \phi_{s2}\left|\mu \right|\phi_{s31}\rangle$	16.296 × 2.967	
p-Br-TRZ	S_25_	S_1_	$\langle \phi_{s0}\left|\mu \right|\phi_{s1}\rangle \times \langle \phi_{s1}\left|\mu \right|\phi_{s25}\rangle$	18.192 × 10.328	5.331 × 10^3^
		S_2_	$\langle \phi_{s0}\left|\mu \right|\phi_{s2}\rangle \times \langle \phi_{s2}\left|\mu \right|\phi_{s25}\rangle$	18.223 × 10.283	

## Data Availability

Not applicable.

## Supplementary Materials

The following supporting information can be downloaded at: https://www.mdpi.com/article/10.3390/molecules28124700/s1, Figure S1. 1,3,5 Triazine derivative molecules with three branched fragments and triazine cores; Figure S2. Heat map of the contribution of molecular fragment in 1,3,5 triazine derivatives to electron and hole contribution of electrons, holes, and electron hole overlap; Figure S3. Scatter plot of the percentage contribution of different fragments of 1,3,5 triazine derivatives to electron excitation, hole and hole overlap; Figure S4. ECD spectrum of o-Br-TRZ, m-Br-TR and p-Br-TRZ. Table S1: Major orbital transition contributions in OPA excited states; Table S2: Interfragment charge transfer and net CT of 1,3,5 triazine derivatives during electron excitation were calculated using the IFCT method.

## Sample Availability

No available.
