# Supplementary material for: Physical Mechanism of One-Photon Absorption, Two-Photon Absorption, and Electron Circular Dichroism of 1,3,5 Triazine Derivatives Based on Molecular Planarity
Source: Molecules. 2023 Jun 11;28(12):4700. doi: 10.3390/molecules28124700 (PMC10305557; doi:10.3390/molecules28124700)
Supplement: Supplementary file 1 [file molecules-28-04700-s001.zip › molecules-2384478-supplementary.pdf]

molecules

# Physical Mechanism of One-Photon Absorption, Two-Photon Absorption, and Electron Circular Dichroism of 1,3,5 Triazine Derivatives Based on Molecular Planarity

**Xiangtao Chen, Xiaoyan Shi, Fuming Yang, Xiqing Zhang, Rui Dai, Yan Jia, Ningte Yan, Sixuan Li, Zihan Wang and Zhongzhu Liang ***

Center for Advanced Optoelectronic Functional Materials Research and Key Laboratory of UV Light-Emitting Materials and Technology of Ministry of Education, College of Physics, Northeast Normal University, Changchun 130024, China; chenxt@nenu.edu.cn (X.C.); shixy@nenu.edu.cn (X.S.); yangfuming18@mails.ucas.ac.cn (F.Y.); zhangxq0723@163.com (X.Z.); dair986@nenu.edu.cn (R.D.); jiay071@nenu.edu.cn (Y.J.); yannt674@nenu.edu.cn (N.Y.); lisx554@nenu.edu.cn (S.L.); wangzh624@nenu.edu.cn (Z.W.)

* Correspondence: liangzz@nenu.edu.cn

## 1. MO transition

**Table S1.** Major orbital transition contributions in OPA excited states.

| Excited energy, Wavelength, Oscillator strength, Spin multiplicity and percentage contribution of orbital transition (>5%) |
|---|
| o-Br-TRZ |
| Excited states: $S_1$ Excited energy :4.081eV Wavelength:281.26nm Oscillator strength :1.3162 Spin multiplicity=1 |
| MO transitions:H→L 27.2%, H-2→L 24.6%, H-1→L+1 20%, H→L+2 6.8% |
| Excited states: $S_2$ Excited energy :4.415eV Wavelength:280.80nm Oscillator strength :1.3588 Spin multiplicity=1 |
| MO transitions:H-2→L+1 27.2%, H-1→L 22.9%, H→L+1 19.8%, H-1→L+2 6.9% |
| m-Br-TRZ |
| Excited states: $S_1$ Excited energy :4.288eV Wavelength:289.12nm Oscillator strength :1.6480 Spin multiplicity=1 |
| MO transitions:H-1→L+1 24.8%, H→L 15.8%, H-2→L+1 13.5%, H-2→L 10.1%, H→L+2 8.3%, H-1→L 8.2%, H→L+1 6.5% |
| Excited states: $S_2$ Excited energy :4.293eV Wavelength:288.82nm Oscillator strength :1.7098 Spin multiplicity=1 |
| MO transitions:H-2→L 30.4%, H→L+1 20.8%, H-1→L 10.8%, H-2→L+1 10.7%, H-1→L+2 7.9% |
| p-Br-TRZ |
| Excited states: $S_1$ Excited energy :4.215eV Wavelength:294.16nm Oscillator strength :1.8720 Spin multiplicity=1 |
| MO transitions:H-2→L 23.4%, H→L+1 15.2%, H-1→L 15%, H→L+2 10.4%, H-2→L+1 8.5%, H→L 8.1%, H-1→L+1 7.2% |
| Excited states: $S_2$ Excited energy :4.216eV Wavelength:294.07nm Oscillator strength :1.8768 Spin multiplicity=1 |
| MO transitions:H-2→L+1 25.4%, H-1→L+1 14.3%, H→L 14.1%, H-1→L+2 10.4%, H-2→L+2 9.4%, H-1→L 7.6% |

## 2. Interfragment charge transfer (IFCT) analysis

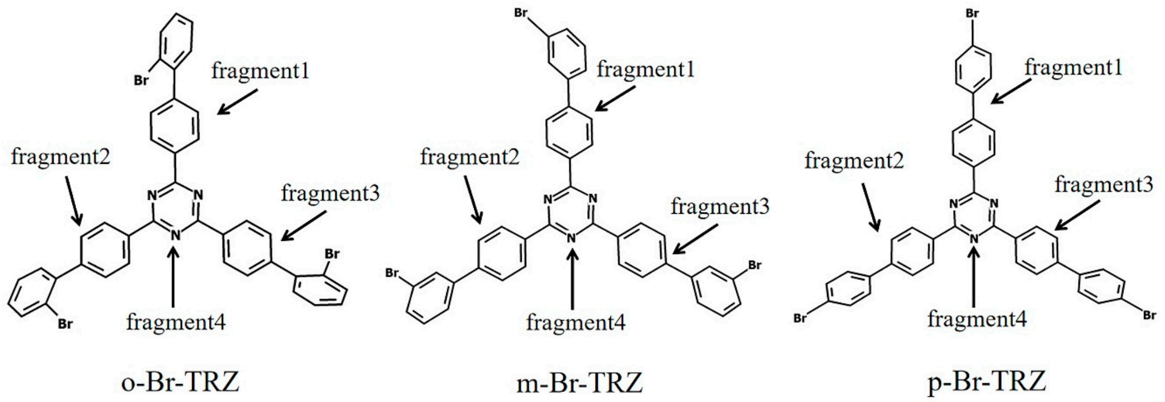


**Figure S1.** 1,3,5 Triazine derivative molecules with three branched fragments and triazine cores.

**Table S2.** Interfragment charge transfer and net CT of 1,3,5 triazine derivatives during electron excitation were calculated by IFCT method.

| Molecule | states | Variation of population number of fragment | | | | Net charge transfer between segments | | |
|---|---|---|---|---|---|---|---|---|
| | | f1 | f2 | f3 | f4 | Net f1→f4 | Net f2→f4 | Net f3→f4 |
| o-Br-TRZ | S1 | -0.1861 | 0.0011 | -0.0756 | 0.2605 | 0.1712 | 0.0116 | 0.0777 |
| | S2 | 0.0050 | -0.1801 | -0.0919 | 0.2670 | 0.0111 | 0.1667 | 0.0891 |
| m-Br-TRZ | S1 | -0.1171 | 0.0152 | -0.1789 | 0.2808 | 0.1118 | 0.0032 | 0.1658 |
| | S2 | -0.0718 | -0.1978 | -0.0075 | 0.2772 | 0.0757 | 0.1798 | 0.0217 |
| p-Br-TRZ | S1 | -0.0705 | -0.0083 | -0.1956 | 0.0386 | 0.0721 | 0.0219 | 0.1803 |
| | S2 | -0.1121 | -0.1743 | 0.0129 | 0.2734 | 0.1106 | 0.1601 | 0.0027 |

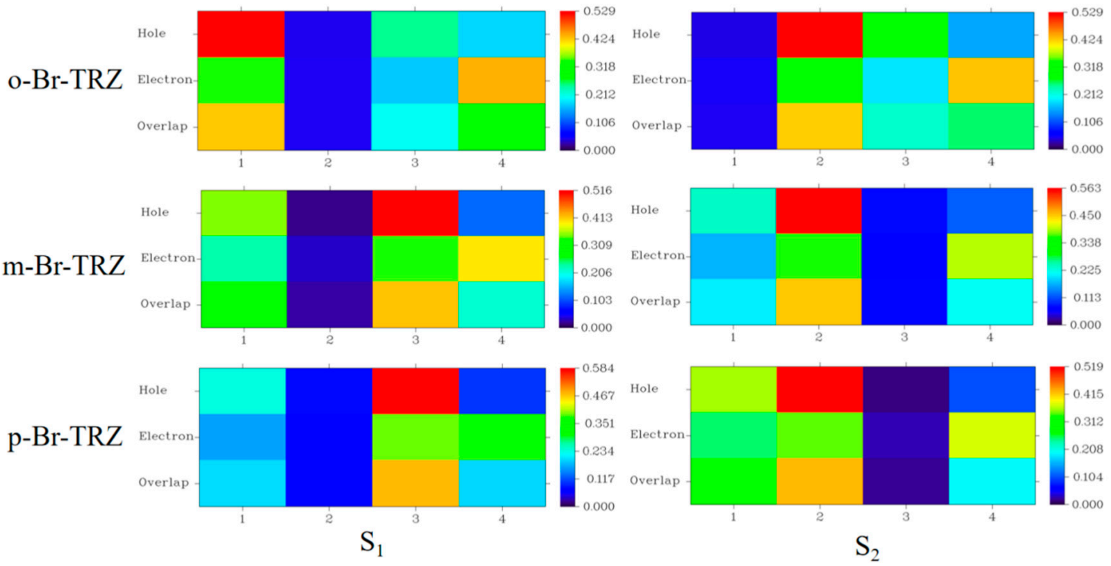


**Figure S2.** Heat map of the contribution of molecular fragment in 1,3,5 triazine derivatives to electron and hole contribution of electrons, holes, and electron hole overlap.

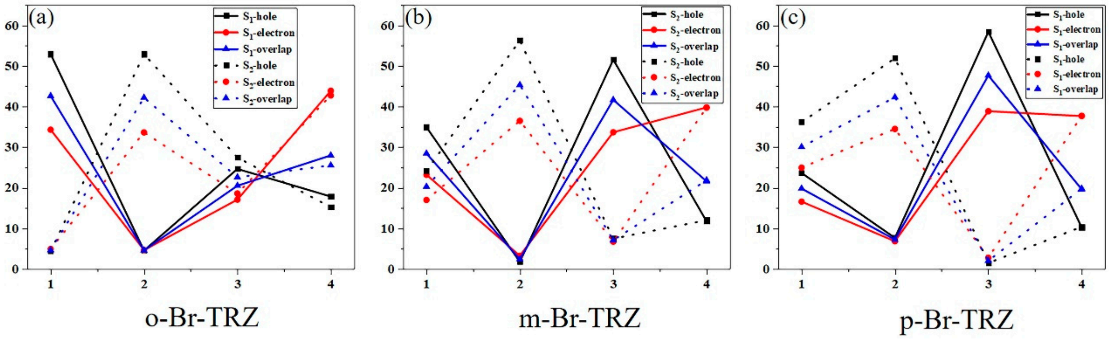


**Figure S3.** Scatter plot of the percentage contribution of different fragments of 1,3,5 triazine derivatives to electron excitation, hole and hole overlap.

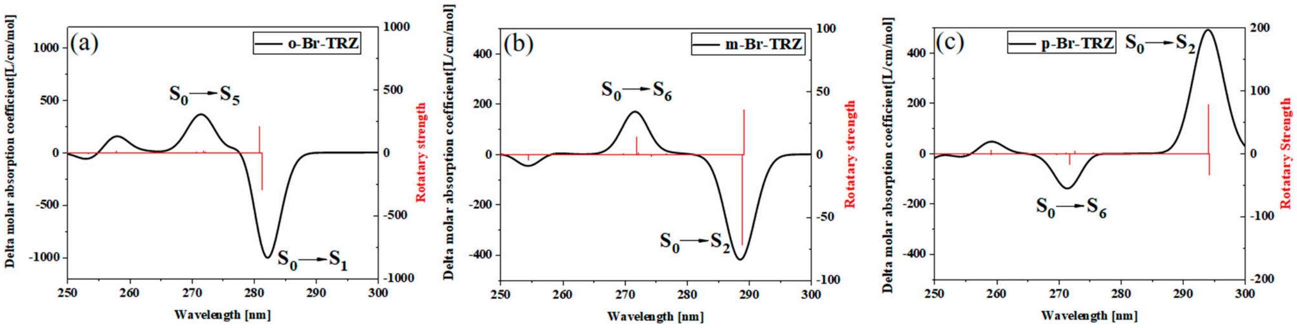


**Figure S4.** (Electron Circular Dichroism) ECD spectrum of o-Br-TRZ, m-Br-TR and p-Br-TRZ.
